# Radiogenic angiosarcoma of the breast: case report and systematic review of the literature

**DOI:** 10.1186/s12885-018-4369-7

**Published:** 2018-04-24

**Authors:** Askin Dogan, Peter Kern, Beate Schultheis, Günther Häusler, Günther A. Rezniczek, Clemens B. Tempfer

**Affiliations:** 1 Department of Obstetrics and Gynecology, Ruhr-Universität Bochum, Bochum, Germany; 2 Department of Obstetrics and Gynecology, St. Elisabeth Hospital, Bochum, Germany; 3 Department of Hematology and Oncology, Ruhr-Universität Bochum, Bochum, Germany; 4 Karl Landsteiner Institute of Gynecological Diagnostics & Therapy, Mauerbach, Austria; 5 Department of Obstetrics and Gynecology, Ruhr-Universität Bochum – Marien Hospital Herne, Hölkeskampring 40, 44625 Herne, Germany

**Keywords:** Ovarian cancer, Endometrial cancer, Synchronous cancer, HNPCC, Young women, Cancer syndrome

## Abstract

**Background:**

Radiogenic angiosarcoma of the breast (RASB) is a rare late sequela of local irradiation of the breast or chest wall after breast cancer. The prognosis of women with RASB is poor and there is no standardized therapy for this type of malignancy.

**Case presentation:**

We present the case of a 54 year old woman with RASB (poorly differentiated angiosarcoma of the left breast; pT1, pNX, M0, L0, V0) and a history of invasive-ductal cancer of the left breast (pT1b, G2, pN0, ER positive, PR positive, HER-2/neu negative) treated in July 2012 with breast-conserving surgery, adjuvant chemotherapy with 6 cycles of epirubicin and cyclophosphamide, adjuvant irradiation of the left breast with 50 Gray, and adjuvant endocrine therapy with an aromatase inhibitor. In August 2016, a bilateral salpingo-oophorectomy was performed to remove a tumor of the left ovary, which was diagnosed as breast cancer recurrence. At the same time, a small, purple skin lesion of 1.2 cm in diameter was noted in the inner upper quadrant of the right breast. RASB was diagnosed by punch biopsy and the tumor was excised with clear margins. Imaging studies showed no evidence of further metastases. A systemic chemotherapy with 6 cycles of liposomal doxorubicin was initiated. Five months later, a local recurrence of RASB was diagnosed and mastectomy was performed. Six months later, the patient is alive with no evidence of disease.

Three hundred seven cases of RASB were identified. The pooled incidence rate of RASB was 1/3754 women. The most common treatment of RASB was mastectomy in 83% of cases. Adjuvant radiotherapy or chemotherapy were rarely used with 6 and 4%, respectively, whereas in case of recurrence, chemotherapy was the mainstay of treatment, used in 58% of cases. Radiotherapy and repeated surgery were also common with 30 and 33% of cases, respectively. Overall, the prognosis of women with RASB was poor and the recurrence-free survival was short with a mean of 15.9 months. Mean overall survival was 27.4 months.

**Conclusion:**

RASB is a rare late complication of breast irradiation. The prognosis of women with RASB is poor. Surgery is the mainstay of treatment for localized disease while systemic chemotherapy and re-irradiation are appropriate for women with disseminated or recurrent RASB.

## Background

Angiosarcoma of the breast is an unusual malignancy accounting for less than 1% of all soft tissue sarcomas [1]. It may develop spontaneously or subsequent to breast irradiation in women after surgical treatment of breast cancer. Sporadic angiosarcoma of the breast is extremely rare. For example, in a Danish region with a population of 1.25 million, Holm et al. identified 42 cases over a period of 35 years for a yearly population-based incidence of 1 in 1 million [2]. In contrast, radiogenic angiosarcoma of the breast (RASB) is much more common among women with a history of breast irradiation. Spontaneous and radiogenic angiosarcomas are morphologically undistinguishable, but there are notable pathogenetic differences. For example, Lae et al. compared the c-myc amplification on chromosome 8q24.21 in 32 RASB specimens and 15 sporadic angiosarcoma specimens [3]. Amplification (5- to 20-fold) of the c-myc oncogene was found in all RASB cases but only in one sporadic angiosarcoma demonstrating a specific oncogenic pathway for RASB. These data also suggest that c-myc may be a potential target for a targeted therapy of RASB.

Typically, RASB is diagnosed as a late sequela of breast irradiation following breast-conserving surgery for invasive breast cancer. In a series of 8 women with RASB treated over a period of 9 years, the cumulative incidence of RASB in the investigated population of women with breast cancer and breast irradiation was 0.14% [4]. Marchal et al. collected follow-up data of 18,115 breast cancer patients from 11 French cancer centers and identified 9 cases of RASB for an incidence of 5/10000 women [5]. In this retrospective case series, the median latency period between breast irradiation and the diagnosis of RASB was 74 (range 57 to 108) months. The prognosis of women with RASB in this case series was poor with 8/9 patients developing early recurrence with a median overall survival time of only 15 months. These data are consistent with a large series of 79 women with RASB from the Memorial-Sloan Kettering Cancer Center demonstrating a high rate of local and distant recurrences and poor survival [6]. In this study, the median interval between breast irradiation and RASB was 7 years and the median time interval between the first treatment of RASB and local or distant recurrence was 1.3 and 2.5 years, respectively. Median survival was 2.9 years. Of note, older age and deep infiltrating RASB were independent predictors of poor survival.

The clinical presentation of RASB is diverse. Lesions are often described as small, purple, teleangiectasia-like formations and may appear as nodules, plaques, or patches [3–6]. RASB often present as multiple, distinct lesions. Thus, a thorough clinical examination is important in order not to overlook satellite lesions. RASB can have different colours, but are mostly described as purple, blue, or black. Another important issue is diagnostic delay. Clearly, RASB is a rare finding and the clinical presentation is uncharacteristic. Therefore, in many patients described in the literature, a substantial time delay between the first notice of the lesion and the final diagnosis of RASB, which requires a histological specimen, has been noted [7, 8].

Due to the rarity of RASB, there is no standardized therapy regimen for women with this disease. Radical surgery of the tumor either by local resection or mastectomy is the most commonly cited treatment [4–6] and complete tumor resection is associated with an improved prognosis. For example, in a series of 21 women with RASB from the Netherlands, Strobbe et al. reported a 2 year overall survival rate of 86% after complete surgical resection compared to 0% after incomplete resection of the tumor [9]. In contrast to the well-established role of surgery, the value of re-irradiation and systemic chemotherapy is less clear. For example, in the series of D’Angelo et al. [6], 78 of 79 women underwent local surgery. Synchronous or metachronous chemotherapy was used in case of unresectable or metastatic disease and was applied to 23 of 79 patients. The most commonly used chemotherapy regimens in this patient cohort were liposomal doxorubicin and paclitaxel. In addition, the mTOR inhibitor sirolimus and targeted therapies such as sorafenib and brivanib were used empirically after chemotherapy had failed. In the treatment of metastatic soft tissue sarcomas and other non-gynecological sarcomas, pazopanib, sirolimus, and brivanib have been described to be active substances after the failure of standard chemotherapy [6, 10, 11]. Re-irradiation or adjuvant chemotherapy were not reported in this patient collective. However, re-irradiation of the breast in women with RASB is feasible and may be associated with a good long-term outcome in selected cases. For example, Smith et al. used hyperfractionated and accelerated re-irradiation with 45 to 75 Gray alone or combined with surgery in 14 women with RASB and reported a median survival of 7 years [12].

To highlight the clinical characteristics, management, and prognosis of women with RASB, we report the case of a woman with histologically verified RASB. In addition, we present a systematic review of the literature with cohort studies, case series, and case reports of women with RASB and discuss the most common therapies and respective outcomes.

## Case presentation

**Fig. 1:**
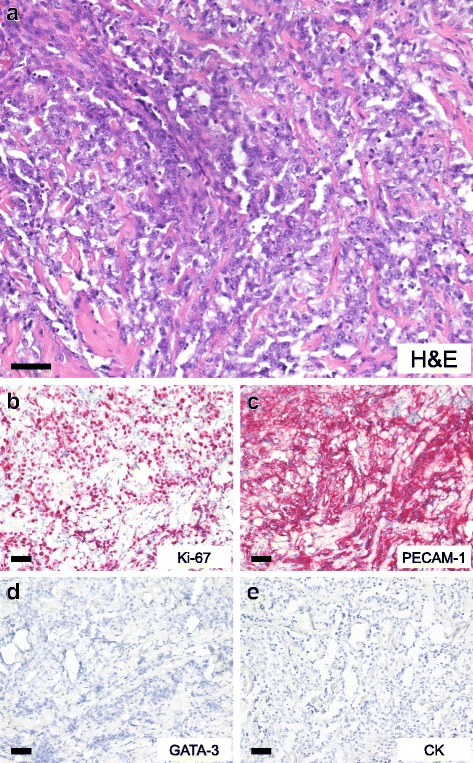
Hematoxylin-eosin (H&E) (**a**) and immunohistochemical (**b**–**e**) stains of a radiogenic angiosarcoma of the breast. The specimen demonstrated expression of proliferation marker protein Ki-67 and platelet endothelial cell adhesion molecule (PECAM-1), but no expression of trans-acting T-cell-specific transcription factor GATA-3 or cytokeratins (CK) was detected. Black bars, 50 μm

**Fig. 2:**
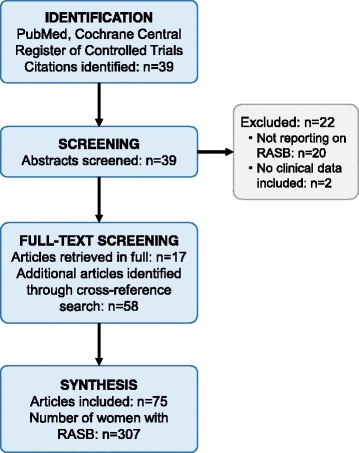
Hematoxylin-eosin (H&E) stain of the initial radiogenic angiosarcoma of the breast

We present the case of a 54 year old woman with RASB (poorly differentiated angiosarcoma of the left breast; pT1a, pNX, M0, L0, V0) after a history of invasive-ductal cancer of the left breast, pT1c (1.8 cm), G3, pN3a (22/23), L1, V0, M0, ER positive, PR positive, HER-2/neu negative) treated in July 2012 with breast-conserving surgery with clear resection margins, adjuvant chemotherapy with 6 cycles of epirubicin and cyclophosphamide, adjuvant irradiation of the left breast and left axillary, supra-, and infraclavicular region with 50 Gray, and adjuvant endocrine therapy with the aromatase inhibitor anastrozole. In August 2016, a bilateral salpingo-oophorectomy was performed to remove a tumor of the left ovary, which was diagnosed as distant breast cancer recurrence. At the same time, in August 2016, a small, purple skin lesion of 1.2 cm in diameter was noted in the inner upper quadrant of the right breast. RASB was diagnosed by punch biopsy and the tumor was completely excised. Staging procedures (computed tomography scans of the thorax and abdomen, bone scintigraphy) showed no evidence of further recurrence. A systemic second-line chemotherapy with 6 cycles of liposomal doxorubicine was initiated. Five months later, a local recurrence of RASB was diagnosed and mastectomy was performed. Staging procedures (computed tomography scans of the thorax and abdomen, bone scintigraphy) were again performed and showed no evidence of distant metastases. No further chemotherapy was applied. After 5 months of follow-up, the patient is alive with no evidence of disease. Figure 1 shows the histological presentation of the RASB in the mastectomy specimen of the left breast as well as immunohistochemical stainings for proliferation marker protein Ki-67, platelet endothelial cell adhesion molecule (PECAM-1), trans-acting T-cell-specific transcription factor GATA-3, and cytokeratins. The tumor demonstrated strong positivity for MIB1 and PECAM-1, but negativity for GATA-3 and cytokeratin. Figure 2 shows an image of the initial RASB diagnosed in August 2016.

In general, angiosarcomas including RASB are high-grade tumors of endothelial origin. They arise from small blood or lymphatic vessels and display varying degrees of nuclear atypia, hyperchromatic nuclei, large nucleoli, and frequent mitoses. Ultrastructural findings may include Weibel-Palade bodies (tubular structures found in normal endothelium) and pinocytic vesicles. Areas of hemorrhage into the surrounding stroma may also be present [1]. Pathology typically shows stratified squamous epithelium and a superficial dermal based proliferation of large, plump, atypical spindled and epithelioid cells with large, pleomorphic vesicular nuclei and prominent nucleoli [5, 10, 13, 14]. Additional typical features including spindle cells with pleomorphic epithelioid eosinophilic cytoplasm and small papillary proliferations.

## Literature review

**Fig. 3:**
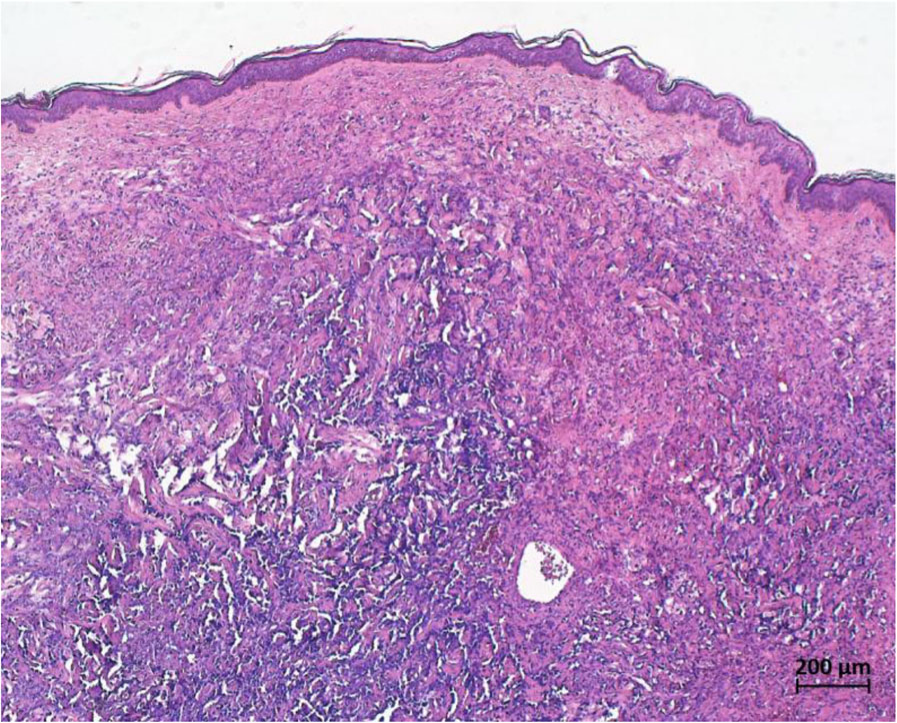
Flow diagram of the literature search algorithm. RASB, radiogenic angiosarcoma of the breast

In a systematic literature search of the databases PubMed and Cochrane Central Register of Controlled Trials (search date 13–04-2017) using the search terms postirradiation[All Fields] AND (“hemangiosarcoma”[MeSH Terms] OR “hemangiosarcoma”[All Fields] OR “angiosarcoma”[All Fields]) AND (“therapy”[Subheading] OR “therapy”[All Fields] OR “treatment”[All Fields] OR “therapeutics”[MeSH Terms] OR “therapeutics”[All Fields]), we identified 39 citations. After screening all abstracts, 17 citations were identified reporting on women with RASB, defined for the purpose of this review as angiosarcoma of the breast after a history of ipsilateral invasive breast cancer and subsequent breast or chest wall irradiation independent of the time interval between breast irradiation and RASB [4, 13–28]. Studies not reporting on women with RASB, double publications, and studies reporting on women with primary angiosarcomas were excluded. The 17 identified studies were retrieved in full and cross reference searching was performed and identified 58 further studies reporting on women with RASB [5–9, 12, 29–80]. Therefore, in summary, 75 studies were analyzed for this review. Figure 3 shows a flow diagram of the literature search algorithm. Among the 75 studies, we found 8 retrospective cohort studies, 18 retrospective cases series, and 49 case reports. No prospectively collected data were identified. Only 5 studies reported on > 10 patients with RASB describing 79 [6], 21 [9], 14 [12], 31 [45], and 27 [64] cases, respectively. Table 1 shows the study characteristics and outcomes of patients with RASB described in all 75 studies. In summary, 307 cases of RASB have been reported in the literature. Seven studies described the patient populations within which RASB cases were identified [5, 9, 19, 21, 24, 45, 53], thus allowing for a calculation of the incidence of RASB. The respective incidences given in these studies were 9/18115 [5], 21/16500 [9], 4/423 [19], 2/5100 [21], 1/3120 [24], 31/220000 [45], and 3/3295 [53] for a pooled incidence rate of 1/3754.

**Table 1:** Clinical studies describing women with radiogenic angiosarcoma of the breast

Author	Year	Study type	Number of cases (n)	Incidence	Single/Multiple lesions (n/n)	Time (m) since breast RXT (n)	Area of initial RXT (n)	Dosage (Gray) at initial RXT (n)
Fodor [4]	2006	CS	8	–	4/4	75	Br (3); Br + Ax (5)	46 (1); 48 (1); 50 (6)
Marchal [5]	1999	COS	9	9/18115	–	74	Br (9)	50 + B (8);45 (1)
D’Angelo [6]	2013	COS	79	–	–	84	–	–
Strobbe [9]	2016	COS	21	21/16500	14/7	74	Br (21)	50 + B (6**);** 44 + B (1);45 + B (1); 46 + B (1);45 + BT (2);50 + BT (10)
Smith [12]	2014	CS	14	–	–	92	Br (14)	60 (14)
Iqbal [15]	2016	CR	1	–	–	–	–	–
Jayalakshmy [16]	2012	CS	2	–	0/2	36;24	–	–
Vertse [17]	2010	CR	1	–	–	120	–	–
Des Guetz [18]	2009	CS	3	–	–	–	–	–
West [19]	2005	COS	4	4/423	2/2	131;109;52;119	Br (4)	45 + B (1);50 + B (3)
Rao [20]	2003	CS	3	–	0/3	168;48;96	Br (3)	40 + B (1);50 (2)
Seo [13]	2003	CR	1	–	1/0	60	Br	50 (1)
Polgár [21]	2001	COS	2	2/5100	1/1	72;100	Br (1);CW (1)	50 (1);45 (1)
Vesoulis [22]	2000	CR	1	–	0/1	96	Br	–
Layfield [23]	1997	CR	1	–	–	–	Br	–
Sole [24]	1996	COS	1	1/3120	–	172	Br	50 (1)
Wijnmaalen [25]	1993	CR	3	–	3/0	66;77;84	Br (1); Br + Ax (1)	45 + B (2);50 + B (1)
Shaikh [14]	1988	CR	1	–	–	48	Br	–
Segal [26]	1988	CR	1	–	–	–	–	–
Otis [27]	1986	CS	2	–	2/0	37;37	CW + SC + IMA (2)	60 (1);50 (1)
Lo [28]	1985	CR	1	–	1/0	72	CW	–
Armengot-Carbó [29]	2012	CR	1	–	0/1	36	CW	–
Fernández Ortega [30]	2006	CS	2	–	1/1	48;53	Br (2)	50 + B (2)
Di Tommaso [31]	2003	CR	1	–	1/0	108	Br	–
Friedrich [32]	1996	CR	1	–	1/0	108	Br	50
Colville [33]	2000	CR	1	–	0/1	92	Br	50
Hogewind [34]	2004	CS	2	–	2/0	84;60	Br (2)	–
Williams [35]	1999	CR	1	–	1/0	44	Br	40 + B
Mermershtain [36]	2002	CR	1	–	0/1	47	Br	50
Deutsch [37]	2003	CR	1	–	1/0	18	Br	50 + B
Pfeiffer [38]	2006	CR	1	–	0/1	72	Br + Ax	40 + B
Griffa [39]	2000	CR	1	–	0/1	–	–	–
Kariniemi [40]	1998	CR	1	–	–	–	–	–
Hildebrandt [41]	2001	CR	1	–	0/1	66	–	–
Esler-Brauer [7]	2007	CR	1	–	1/0	60	–	–
Catena [42]	2006	CR	1	–	0/1	57	Br	50 + B
Moe [43]	2007	CR	1	–	0/1	30	Br	45
Lamblin [44]	2001	CS	4	–	–	–	–	–
Hodgson [45]	2007	COS	31	31/220000	15/16	62	–	–
Weed [46]	2008	CR	1	–	–	–	–	–
Bolin [47]	1996	CR	1	–	–	120	–	–
Soldić [48]	2009	CR	1	–	1/0	40	–	45 + B
Horevoets [49]	2013	CR	1	–	1/0	–	–	–
Andrews [50]	2010	CR	1	–	0/1	51	MSBT	34
Kajo [51]	2007	CR	1	–	1/0	96	–	50 + B
Nambisan [52]	2008	CR	1	–	–	–	–	–
Zucali [53]	1994	COS	3	3/3295	3/0	18;59;41	Br (3)	50 + B (2); 45 + B (1)
Aydogdu [54]	1996	CR	1	–	0/1	60	–	–
Del Mastro [55]	1994	CR	1	–	–	40	–	–
Weber [56]	1995	CS	3	–	2/1	60 (2);72	Br + Ax (1);Br + Ax+SC + IMA (1);Br + IMA + SC (1)	50 + B (1);50 (2)
Majeski [57]	2000	CR	1	–	0/1	63	Br	50
Wiebringhaus [58]	2015	CS	3	–	2/1	72;120;36	Br (3)	–
Bonetta [59]	1995	CR	1	–	1/0	62	Br	–
Nakamura [60]	2007	CR	1	–	0/1	120	Br	50 + B
Anania [61]	2002	CR	1	–	1/0	120	Br	–
Barbosa [62]	2015	CR	1	–	1/0	120	Br	–
Nestle-Krämling 622]	2011	CS	4	–	1/0	120	Br	–
Billings [64]	2004	CS	27	–	11/13^a^	66	Br (27)	47 + B (11);50 (2);45 (2)^b^
Sessions [65]	1992	CR	1	–	–	54	Br	–
Zafar [66]	2012	CR	1	–	0/1	72	Br	50
Navarro Cecilia [67]	2015	CR	1	–	0/1	84	Br	50 + B
Uryvaev [68]	2015	CS	6	–	5/1	110	Br (5);Br + Ax (1)	50 + B (1);61 (1);45 (1);40 + B (1);50 + B (1)^c^
Gennaro [69]	2010	CS	9	–	–	96	–	–
Feigenberg [8]	2002	CS	3	–	0/3	48 (2);60	Br	50 (1);50 + B (2)
Hui [70]	2012	CS	8	–	–	84 (8)	–	–
Taat [71]	1992	CR	1	–	–	66	Br	–
Adhikari [72]	2002	CR	1	–	1/0	204	Br	50
Azizun-Nisa [73]	2013	CS	3	–	2/1	–	–	–
Hanasono [74]	2005	CR	1	–	0/1	80	CW + SC	–
Mocerino [75]	2016	CR	1	–	1/0	94	Br	60
Scow [76]	2010	CR	1	–	1/0	94	Br	60
Zemanova [77]	2014	CR	1	–	0/1	144	Br	46 + B
Moskaluk [78]	1992	CR	1	–	1/0	96	Br	20 + BT
Plichta [79]	2017	CR	1	–	0/1	60	Br	–
Boyan [80]	2014	CR	1	–	0/1	60	Br	–
Pooled Analysis	–	–	307	1/3754	87/75	77.2	–	–

*Abbreviations*: *Ax* axilla, *B* boost, *Br* breast, *BT* brachytherapy, *COS* cohort study, *CR* case report, *CS* case series, *CW* chest wall, *IMA* internal mammary lymph nodes, *m* months, *MSBT* Mammosite® brachytherapy, *n* number of cases, *RXT* radiotherapy, *SC* supraclavicular lymph nodes, *TCG* telecobalt-gamma

^a^3 cases unknown

^b^12 cases unknown

^c^1 case unknown

At the time of first presentation, single or multiple local lesions were described in 162 cases. Single and multiple lesions were evenly distributed with 87 and 75 cases, respectively. The mean time between breast cancer irradiation and the diagnosis of RASB was 77.2 months.

Treatment modalities and outcomes are shown in Table 2. The most commonly used treatment of RASB was mastectomy reported in 85% of cases. Adjuvant chemotherapy or adjuvant irradiation was rarely given with 4 and 6% of cases reported in the pooled analysis. After recurrence of RASB, however, chemotherapy was most often used with 58% of cases. There is no standard chemotherapy regimen for RASB, but anthracyclines and taxanes were the most commonly used substances. In addition, ifosfamide, doxorubicine, and gemcitabine were reported alone or in combination with anthracyclines and taxanes. Irradiation and repeated surgery were also common treatment modalities in women with recurrent RASB and have been reported in 30 and 33% of cases, respectively. Overall, the prognosis of women with RASB was poor. The recurrence-free survival was short with a mean of 15.9 months and overall survival was 27.4 months.

**Table 2:** Treatment modalities and outcomes of women with radiogenic angiosarcoma of the breast

Author	Year	Treatment modalities (Initial)	Treatment modalities (Recurrence)	Chemotherapy (n)/ regimen	RXT (n)/ gray	Recurrence-Free survival (m)	Overall survival (m)
Fodor [4]	2006	MAS (8)	S + CHXT (1)	1/DOCE	–	16	26
Marchal [5]	1999	MAS (4);MAS + CHXT (2); MAS + RXT (2);MAS + S (1)	S (1);RXT; CHXT (7)	2/−	3/30;45^a^	7	15
D’Angelo [6]	2013	MAS (65);S (13)^a^; CHXT (9)	CHXT (23)	23/DOX (9), PAC (3), SORA (3), BRIV (2), IFO (1), DOX + PAC (1), GEM+DOCE (1), SIR (1)	–	16	36
Strobbe [9]	1998	MAS (20);MAS + S (1)	S (7)	–	–	13	27
Smith [12]	2014	MAS (6);MAS + RXT (8)	RXT (6)	–	14/45 (2);60 (3);75 (9)		97
West [19]	2005	MAS (2);MAS + RXT (1);MAS + CHXT (1)	S (1); CHXT (1)	2/TAX;ADR	2/−	26;6;3;5	26;19;12;6
Rao [20]	2003	MAS (3)	S + CHXT (1)	–	–	41;3;7	41;12;7
Polgár [21]	2001	MAS (1);NT (1)	MAS (1)	–	–	36;4	36;4
Vesoulis [22]	2000	MAS + CHXT (1)		1/−	–	–	–
Wijnmaalen [24]	1993	MAS (2);MAS + S (1)	S (1)	–	–	7;16;30	7;30;34
Otis [27]	1986	S (2)	–	1/−	–	2	–
Armengot-Carbó [29]	2012	MAS (1)	–	–	–	9	9
Fernández [30]	2006	MAS (2)	S (1)	–	–	8;50	10;50
Colville [33]	2000	S (1)	MAS;S;RXT (1)	–	1/40	8	20
Hogewind [34]	2004	MAS (2)	S (1)	–	–	4;24	4,24
Williams [35]	2004	MAS (1)	–	–	–	5	5
Deutsch [37]	2003	MAS + S (1)	S;RXT (1)	–	1/−	4	23
Hildebrandt [41]	2001	MAS (1)	S (1)	–	–	16	23
Esler-Brauer [7]	2007	MAS (1)	–	–	–	45	45
Catena [42]	2006	MAS (1)	–	–	–	10	10
Moe [43]	2007	MAS (1)	S;CHXT (1)	1/DOX;IFO	–	2	20
Hodgson [45]	2007	MAS (25)		–	–	–	–
Soldić [48]	2009	MAS	–	–	–	16	16
Zucali [53]	1994	S (1);MAS (2); MAS + S (1)	S + RXT (1);CHXT (1)	1/−	1/60	96;4;13	96;7;24
Aydogdu [54]	1996	MAS (1)	–	–	–	3	–
Weber [56]	1995	S (2);RXT + CHXT (1)	RXT;CHXT (1)	1/5-FU, DOX	–	36;24	36;24
Majeski [57]	2000	MAS + S (1)	S (1)	–	–	26	29
Nakamura [60]	2007	MAS + CHXT (1)	–	1/PAC	–	15	15
Billings [64]	2004	MAS (10); S (10)^a^	CHXT (4)	4/−	–	–	42
Navarro Cecilia [67]	2015	MAS (1)	S (1)	–	–	1	7
Uryvaev [68]	2015	MAS (3);MAS + RXT (3)	RXT (2);CHXT (3)	3/ADR + IFO (1); DOX + PAC (2)	3/50 (2);16 (1)	–	43
Gennaro [69]	2010	MAS (9)	–	5/ADR + IFO	–	–	–
Feigenberg [8]	2002	MAS (3)	RXT (3)	–	3/60 (2);50 (1)	2;1;22	41;60;22
Hui [71]	2012	MAS (1);MAS + RXT (4);MAS + CHXT (3)	–	3/PAC	–	–	–
Adhikari [72]	2002	S + RXT + CHXT (1)	S (1)	1/ADR + CYC	1/50	13	16
Hanasono [74]	2005	S (1)	S,CHXT (1)	–	–	2	13
Mocerino [75]	2016	MAS (1)	S;CHXT;RXT (1)	1/BLEO;DOX	1/60	12	24
Moskaluk [78]	1992	S (1)	MAS (1)	–	–	24	96
Pooled Analysis	–	MAS (83%); S (13%); CHXT (4%) (adjuvant); RXT (6%) (adjuvant)	CHXT (58%); S (33%); RXT (30%)^**b**^	–	–	15.9	27.4

*Abbreviations*: *5-FU* 5-fluorouracil, *ADR* adriamycin, *BLEO* bleomycin, *BRIV* brivanib, *CHXT* chemotherapy, *CYC* cyclophosphamide, *DOCE* docetaxel, *DOX* doxoribicin, *GEM* gemcitabine, *IFO* ifosfamide, *m* months, *MAID* doxorubicin, ifosfamide, dacarbazine, *MAS* mastectomy, *n* number, *NT* no treatment, *PAC* paclitaxel, *RXT* radiotherapy, *S* surgery, *SIR* sirolimus, *SORA* sorafenib

^a^Missing cases not documented

^b^multiple therapies possible

The largest cohort of women with RASB was published by D’Angelo et al. [6]. The authors described 79 women with RASB treated at the Memorial Sloan Kettering Cancer Center between 1982 and 2011. In this study, the diagnosis of RASB was defined pathologically by the presence of solid growth with variable angioformative features and overt cytologic atypia. Almost all women were initially treated with surgery, either mastectomy (65 cases) or local excision (13 cases). Complete RASB resection with free resection margins was achieved in 52/79 (66%) of cases. Follow-up data were available for 65 women, of whom 37 (60%) were still alive after a median follow-up of 4.5 years. The importance of radical initial surgery was underlined by a univariate analysis demonstrating that resection margin status was the single most important prognostic factor regarding distant recurrence-free survival. Older age and deeply infiltrating RASB (as opposed to superficial disease) were independent prognostic factors for disease-specific survival.

Three other large patient cohorts including 31 [45], 27 [64], and 21 [9] cases of RASB were identified. In line with the data of D’Angelo et al., Hodgson et al. reported mastectomy as the treatment of first choice in 81% and an overall mortality of 58% [45]. In a series of 27 cases, Billings et al. found that the median interval between breast irradiation and the diagnosis of RASB was 59 months [64]. Only in 5 women RASB occurred within less than 3 years after breast irradiation. Lymphedema was not a typical presentation of RASB. Multifocal appearance at first diagnosis was noted in half of the cases (13 of 27 cases) and all tumors had a vasoformative growth pattern. Other features characteristic for the histological appearance of RABS were a sieve-like pattern and high-grade nuclear features, whereas necroses were rare. All women were initially treated surgically with wide excision or mastectomy. Fourteen women experienced local recurrence and 6 had multiple recurrences. Metastasis was documented in 9 of 22 patients, 8 of whom died of the disease. Strobbe et al. collected data on 21 RASB cases diagnosed in the Netherlands between 1987 and 1995 [9]. In this series, the median interval between breast irradiation and RASB was 74 months and appeared to decrease with higher age. The 2 year overall and disease free survival rates were 72 and 35%, respectively. In accordance with the previously cited studies, complete initial resection of RASB was also an important prognostic factor with a 2 year overall survival rate after initial complete surgical resection of 86% compared to 0% after incomplete resection.

The bulk of studies identified in this systematic review were small case series and case reports [7, 8, 13–44, 46–63, 65–67, 70, 72–80]. As expected, the heterogeneity among these studies with low numbers of RASB patients was considerable. However, as shown in Table 2, most patients were treated with mastectomy, whereas adjuvant chemotherapy and radiotherapy were rarely used. Long-term survivors among these women were only found in cases of localized disease at initial presentation and complete tumor resection.

## Discussion

In this case report and systematic review of the literature, we found that RASB is a rare late complication of breast irradiation and carries a poor prognosis. Surgery is the mainstay of treatment for localized disease while systemic chemotherapy and re-irradiation are appropriate for women with disseminated or recurrent RASB. Specifically, we identified and analyzed 307 cases of RASB for a pooled incidence rate of RASB of 1/3754 women after breast irradiation. Overall, the prognosis of women with RASB is poor and the reported mean recurrence-free survival is only 15.9 months. Mean overall survival is 27.4 months.

Chemotherapy, although only rarely used in the adjuvant setting, is used in the majority of cases after recurrence. In our pooled analysis, chemotherapy was given in 58% of women with recurrent RASB. The choice of drug regimen, clearly, was empirical due to the rarity of the disease and there is no standard chemotherapy regimen for RASB. Among the many regimens and substances, anthracyclines and taxanes were the most commonly used compounds. In addition, ifosfamide and gemcitabine have been used alone or in combination with anthracyclines and taxanes. This is in accordance with what has been reported in the treatment of patients with metastatic angiosarcoma. For example, in a large series of D’Angelo et al., including 119 cases of metastatic angiosarcomas [81], doxorubicin and taxanes were the most commonly used agents. The overall response rate to first line chemotherapy was 30% and below 10% in all subsequent chemotherapy lines. The median time to progression was 3.5 months for first line chemotherapy, 3.7 months for second line chemotherapy, and 2.7 months for third line chemotherapy. Of note, both anthracyclines and taxanes resulted in similar response rates and survival times. In addition, there was no apparent benefit for combination chemotherapy regimens compared to single agents. These results are in accordance with another analysis of chemotherapy efficiency among 117 cases with metastatic angiosarcomas published by Italiano et al. [82]. In this study, weekly paclitaxel (used in 64% of cases) and doxorubicin (used in 36% of cases) were again the most commonly used agents. Both had similar efficacy. In the doxorubicin group, 2 (6%) had complete response, 8 (23.5%) had partial response, 10 (29.5%) had stable disease, and 14 (41%) had progressive disease. In the paclitaxel group, 9 (13%) had complete response, 27 (40%) had partial response, 20 (29.5%) had stable disease, and 12 (17.5%) had progressive disease. Objective responses to weekly paclitaxel were more frequent in cutaneous angiosarcomas, whereas tumor location did not impact response to doxorubicin. Median progression-free survival was 4.9 months and median overall survival was 8.5 months. The dosage and therapy duration of regimens including taxanes and anthracyclines varied in the literature. For example, Italiano et al. used single-agent doxorubicin in a dosage of 60–75 mg/m2 on day 1 in a 3-week cycle or weekly paclitaxel at a dosage of 80 mg/m2/day in the first-line setting [82]. Others used polychemotherapy regimens with 6 cycles and the following dosages: cyclophosphamide 500 mg/m2, vincristine 1.4 mg/m2, doxorubicin 50 mg/m2 on day 1, and dacarbazine 400 mg/m2 on days 1 to 3 with cycles repeated every 28 days or doxorubicin 60 mg/m2 on day 1, ifosfamide 2.5 g/m2 on days 1 to 3, and dacarbazine 800 mg/m2, given on day 2, cycles repeated every 28 days or doxorubine 60 mg/m2 and cisplatin 100 mg/m2 on day 1 and ifosfamide 3 g/m2 on days 1 to 3, repeated every 28 days [18].

It is of note that angiogenesis inhibitors such as the vascular endothelial growth factor (VEGF) antibody bevacizumab have not been used in women with RASB, although this would be a logical treatment approach. On the other hand, bevacizumab has not proven efficacious in preliminary studies in women with metastatic sporadic angiosarcomas. For example, Ray-Coquard performed a randomized phase II trial and reported no additional benefit regarding progression-free survival and overall survival when adding bevacizumab (10 mg/kg once every 2 weeks) to weekly paclitaxel with 90 mg/m2 [83].

Another targeted substance with a rationale for the treatment of RASB is pazopanib, an antiangiogenic drug, which has been successfully used in angiosarcomas as well as other sarcoma entities, e.g. synovial sarcoma [84]. However, no data on the use of pazopanib in women with RASB are available.

The case report presented within this review had typical characteristics of RASB, for example the long time delay between the radiotherapy and the development of RASB. We chose complete local excision as the primary means of therapy based on our consultation of the literature. For example, in our literature review, 85% of RASB patients underwent mastectomy. In addition, we treated the patient with liposomal doxorubicine, because anthracyclines are active substances both in breast cancer and RASB. After the first recurrence of RASB, we performed mastectomy and suggested another line of chemotherapy, which was declined by the patient. Clearly, other therapy options such mTOR inhibitors or targeted therapies such as sorafenib and brivanib could have been used in this patient. However, she declined additional systemic therapies and thus opted for clinical follow-up.

## Conclusions

In conclusion, we found that RASB is a rare late complication of breast irradiation. The prognosis of women with RASB is poor. Surgery is the mainstay of treatment for localized disease while systemic chemotherapy and re-irradiation are appropriate for women with disseminated or recurrent RASB.

## Abbreviations

ER: Estrogen receptor
HNPCC: Hereditary non-polyposis colon cancer syndrome
mTOR: Mammalian target of rapamycin
PECAM: Platelet endothelial cell adhesion molecule
PR: Progesterone receptor
RASB: radiogenic angiosarcoma of the breast
VEGF: Vascular endothelial growth factor
